# Granuloma formation and tissue pathology in *Schistosoma japonicum* versus *Schistosoma mansoni* infections

**DOI:** 10.1111/pim.12778

**Published:** 2020-08-18

**Authors:** Felix Llanwarne, Helena Helmby

**Affiliations:** ^1^ Department of Infection Biology Faculty of Infectious and Tropical Disease London School of Hygiene and Tropical Medicine London UK

**Keywords:** cytokines, granuloma, helminth, neutrophils, schistosome

## Abstract

Schistosomiasis is the most important helminth disease in the world from a public health perspective. *S mansoni* and *S japonicum* account for the majority of global intestinal schistosomiasis cases, and the pathogenesis is widely assumed to be fundamentally similar. However, the majority of research on schistosomiasis has been carried out on *S mansoni* and comparisons between the two species are rarely made. Here, we will discuss aspects of both older and recent literature where such comparisons have been made, with a particular focus on the pathological agent, the host granulomatous response to the egg. Major differences between the two species are apparent in features such as egg production patterns and cellular infiltration; however, it is also clear that even subtle differences in the cascade of various cytokines and chemokines contribute to the different levels of pathology observed between these two main species of intestinal schistosomiasis. A better understanding of such differences at species level will be vital when it comes to the development of new treatment strategies and vaccines.

## 1 INTRODUCTION

Schistosomiasis (bilharzia) is a disease that currently afflicts over 200 million people worldwide. Twenty million individuals are characterized as having severe morbidity, and death estimates vary between 24 000 and 200 000 deaths per year.^1^, ^2^ The disease is endemic in 74 countries across the world and is caused by six species of schistosomes: *Schistosoma mansoni* (occurring in Africa, South America, the Caribbean and the Middle East), *S haematobium* (mainly occurring in Africa and the Middle East), *S intercalatum* and *S guineensis* (confined to a few countries in Central Africa), *S mekongi* (Mekong Delta, including Cambodia and Lao) and *S japonicum* (Asia).^3^ Of these species, *S haematobium* is the principal cause of urinary schistosomiasis, with the remaining giving rise to the intestinal forms of the disease. This heterogeneity of disease outcome is primarily due to the venule location in which the adult worms reside.^3^ Although *S mansoni* is the most common intestinal species across Africa and Latin America, in Asia *Schistosoma japonicum* is the most prevalent species. While China is making good progress in controlling *S japonicum,* ^4^ it remains a major health risk in other parts of Asia, most notably in the Philippines where *S japonicum* is moderately endemic (5‐25%) in most of the endemic regions.^5^, ^6^ Complicating this is the fact that *S japonicum* is a zoonosis and can infect a wide array of animals such as dogs,^7^ wild rats, cattle, and in particular, water buffaloes.^8^ No vaccine is available against any species of schistosome but considerable progress in the control of schistosomiasis has been made in recent decades, mainly through mass drug administration (MDA) programmes, using Praziquantel administration to entire at‐risk populations without prior diagnosis. However, preventive chemotherapy alone is insufficient to break the transmission cycle and low‐grade chronic infections leading to organ damage, anaemia, growth retardation and cognitive impairment are still prevalent across the world.

The infection is mediated by the freshwater snail intermediate host, with humans acquiring the infection by water contact. Regarding intestinal schistosomiasis, once the parasite is established in the mesenteric veins, the adult female worm produce eggs, half of which exit the host via the intestinal lumen, while half become trapped in the intestinal wall or in organs such as the liver, generating a potent granulomatous inflammatory response leading to hepatosplenomegaly and pipestem fibrosis. The most significant lesion in severe chronic infections is marked periportal fibrosis of the liver (clay pipestem fibrosis or Symmers’ fibrosis) associated with splenomegaly and portal hypertension.

Evolutionarily, *S mansoni* and *S japonicum* are genetically distinct, as highlighted by geographic separation and snail host variation. Since the full genome sequencing of both species in 2009,^9^, ^10^ this differentiation has been further elucidated from combinational sequencing of nuclear and mitochondrial genome sequencing. The prevailing theory is that Central and SE Asian schistosome species (the *S japonicum* group) are phylogenetically basal and separate from all other animal and human *Schistosoma* spp., including the *S mansoni* group. Current estimates place African schistosome colonization 15‐20 million years ago (MYA), *S japonicum*‐*S mansoni* divergence ~14 MYA, and *S mansoni*‐*S haematobium* divergence ~4 MYA,^11^ which together has formed the basis of the ‘out of Asia’ hypothesis regarding the Asian origin of these species.^12^

*S mansoni* and *S japonicum* account for the majority of global intestinal schistosomiasis cases, and the pathogenesis is generally viewed to be fundamentally similar. However, the majority of research on schistosomiasis has been carried out on *S mansoni*, and direct comparisons between the two species are rarely made. Here, we will discuss the aspects of both older and recent literature where such comparisons have been made, with a particular focus on the pathological agent, the host granulomatous response to the egg.

## 2 LARVAL MIGRATION

Already upon initial infection of the schistosomula through the skin, there are marked differences between *S mansoni* and *S japonicum*. Experimental comparisons using human skin cultures found that 90% of *S mansoni* schistosomula were located in the epidermis after 24 hrs, moving on to the dermis after 48 hrs, and by 72 hrs they had reached the dermal vessels. Conversely, 90% of *S japonicum* schistosomula had reached the dermis within 24 hrs post‐infection (p.i.) with some schistosomula found in the dermal vessels already after 2 hrs, demonstrating a much more rapid migration of *S japonicum*.^13^ The skin cytokine expression during *S mansoni* schistosomula migration was dominated by interleukin‐1 receptor antagonist (IL‐1ra), IL‐10 and tumour necrosis factor (TNF)‐α at 8 hrs p.i., while expression of skin cytokines during *S japonicum* migration was seen to be more extensive, consisting of IL‐1β, IL‐1ra, IL‐2, IL‐6, IL‐8, IL‐10, IL‐15, IL‐18 and TNF‐α after 8 hrs, suggesting a broader activation of immune cells during *S japonicum* migration.^13^ In addition, in *S japonicum* infection, there was a marked increase in IL‐1ra in the skin comparative to *S mansoni*, indicative of smokescreen activity subduing inflammation around the parasite to evade detection.^13^

Both species then use the blood circulation and lymphatic system to reach the lungs where *S mansoni* numbers peak after 6 days,^14^ while *S japonicum* numbers peak already after 3 days.^14^, ^15^ The mechanism of *S japonicum's* swift migration is not fully understood; however, it may be that they possess more potent penetrating enzymes through skin tissue, which may also play a part in explaining why this species can establish in over 40 known mammalian host species,^13^, ^16^ while *S mansoni* exhibit a much more limited host specificity.

These differences in immunomodulatory and migratory capabilities between the two species may attribute to variations in the priming of the immune system of naïve individuals and influencing the downstream response to the parasite culminating in acute infection.

## 3 ADULT WORM LOCATION AND OVIPOSITION PATTERNS

While both *S mansoni* and *S japonicum* adult worms primarily occupy the mesenteric veins surrounding the intestines neither species is constrained to one location and appear to move considerable distances through the mesenteries.^17^ Adult *S japonicum* in the portal venous system have been found to aggregate to other worms, forming clusters and creating large areas of focussed pathology around the intestine with adjacent regions relatively free of eggs.^18^ *S mansoni* lesions on the other hand have a more diffuse pattern of distribution, contrasting the clustered, massed or ‘nested’ pattern seen in *S japonicum*.^19^

As the eggs are released from the adult worms, the host response to them dictates the extent of the resulting pathology. As such, any differences between species here will manifest in the attributed diseases. Most notably there are significant differences in egg production rates with *S mansoni* producing around 800 eggs per day per female worm laid as single eggs, whereas *S japonicum* females produce up to 3000 eggs per day laid in clusters of as many as 50.^20^, ^21^ Combined with the clustering of adult *S japonicum* worms in groups, this worm behaviour contributes greatly to the increased disease severity seen in *S japonicum* compared to that of *S mansoni* infections.^19^

## 4 HOST IMMUNE RESPONSE AND LOCAL PATHOLOGY

As with other helminth infections, the host immune response during schistosomiasis infection is characterized by a T helper type 2 (Th2)–skewed response in terms of cytokine profile and activated cell types. There is, however, also a role for Th1 cell activity and cytokines, and the balance between the types of T helper cell responses is strongly regulated over the duration of infection, and a too robust response of either type can result in extensive tissue damage.^22^, ^23^, ^24^, ^25^, ^26^

The initial host immune response after infection is primarily Th1‐mediated, targeting the schistosomulum or juvenile worm and characterized by the production of cytokines such as IL‐1, IL‐12, TNF‐α and interferon (IFN)‐γ.^27^ The host response progressively switches to a Th2‐mediated response around six to eight weeks p.i. as egg deposition begins, characterized by an increase in IL‐4, IL‐5, IL‐13 and the production of immunoglobulin (Ig)E.^28^ Over time, this local Th2 environment leads to the formation of cellular infiltrates (granulomas) and the generation of irreversible tissue fibrosis. The egg is therefore the primary pathogenic factor in schistosomiasis as it acts as the initiator of the granuloma formation and is essential for the pattern of events that lead to the pathology associated with chronic infection.

The granulomatous response to schistosome infections varies temporally but consists of the recruitment of T and B cells, macrophages, neutrophils, myofibroblasts/hepatic stellate cells (HSCs), eosinophils and epithelioid cells.^29^ The cells surround the eggs, forming a wall, and as the granuloma develops, fibrosis and collagen are deposited from activated cells and remain permanently in the tissue even after the destruction of the egg itself. The formation of fibrosis around the eggs is widely detrimental to the host in that it leads to hepatic and periportal fibrosis, resulting in blockage of the blood flow leading to portal hypertension and portal shunting that may result in fatal oesophageal bleeding.^30^ However, the formation of granulomas is also vital in order to limit the egg‐antigen induced inflammation that would otherwise lead to permanent inflammatory tissue damage and extensive necrosis, as seen in the increased mortality in *S mansoni*‐infected T cell–depleted mice.^31^ Thus, although the formation of the schistosome granuloma is the key pathology in schistosomiasis, it is ultimately also beneficial to the host.

The schistosome egg granuloma have been studied extensively since the von Lichtenberg experiments in the 1960s, where sensitization in mouse models decreased egg destruction and accelerated immune responses in terms of relative size of granuloma and percentage of eggs with reactions, which could be transferable by cells and not serum.^32^, ^33^, ^34^ There is a general correlation of granuloma formation with egg maturation over its ~3‐week viable lifespan, and the basic immunopathologic outcomes associated with granuloma formation and fibrosis are generally fairly similar between *S mansoni* and *S japonicum*.^19^

However, key differences between the two species lie in the severity of the pathology and some of the mechanisms underlying the immunologic reactions. Early experiments determined that in *S mansoni* infections, a cell‐mediated delayed type hypersensitive reaction (DTH—also termed type IV hypersensitivity reactions) was elicited in the granulomatous reaction, whereas in *S japonicum* infections DTH responses were actively suppressed,^35^ and instead elicited an immediate type hypersensitive immune response forming granulomas akin to those in foreign body responses, not affected by sensitization and consistently smaller than those of *S mansoni*.^32^ This difference suggests there are key alternative pathways that lead to granuloma formation and hepatic fibrosis in the two species.^29^

The immune reactions to *S japonicum* eggs in hamsters showed consistently smaller granulomas around single eggs but larger around aggregated eggs, and liver enlargement was greater in *S japonicum* chronic infections than in *S mansoni*‐infected livers, possibly due to higher antigen concentrations.^19^ It is argued whether granuloma measurements are completely relevant parameters as they do not account for cell damage or haemorrhaging; however, at all stages of *S japonicum* infections in these early experiments, there was considerably more parenchymal liver damage than in *S mansoni*‐infected tissue pathology.^19^ Moreover, it has generally been found that at comparable egg loads and time spans, the liver pathology of *S japonicum* is the more severe of the two. This is not due to the size of the granulomas formed, but the tendency for *S japonicum* to produce more exudation and necrosis in early granuloma formation, and encroach on adjacent liver tissue with greater diffuse inflammation.^19^ Early experiments found that in *S japonicum* rabbit infections, cirrhosis was out of proportion to the number of eggs deposited.^36^ Moreover, *S japonicum* hamster hepatic pathology displayed abundant dying liver cells or eosinophil‐central necrotic areas in the exudative granulomas or pseudoabscesses and that the epithelioid cell layer demarcating the granulomas was completely absent or poorly developed.^19^ At 1 week post‐patency, *S japonicum* granulomas were surrounded by inflammatory structures, focal haemorrhaging, oedema or stellate fibroblasts and capillary buds which encroached upon neighbouring liver tissue that often exhibited necrosis. Zonal infarct‐like eosinophilic liver necrosis and Councilman bodies (acidophilic aggregates of cells composed of dying hepatocytes often surrounded by normal parenchyma) were frequent. At 11 weeks post‐patency, however, granulomas were delimited by fibroblastic rims and were less encroaching. But at all stages, *S japonicum* had substantially more parenchymal liver disturbance than *S mansoni*. Thus, although single *S japonicum* eggs and granulomas were smaller than those of *S mansoni*, greater liver enlargement was observed and the ratio of hepatic inflammatory tissue to parenchymal liver tissue was higher in individuals infected with *S japonicum*.^19^ Similar *S japonicum* destructive effects have been described in several animal models.^36^, ^37^, ^38^, ^39^ Hence, it is clear that the egg‐induced tissue inflammatory responses and resulting damage is greater in *S japonicum* than in *S mansoni* infections, even at comparable levels of egg deposition.^36^, ^37^, ^38^, ^39^

## 5 COMPOSITION OF THE GRANULOMA

CD4^+^ Th1 cells are involved in the initial granuloma development in *S mansoni* and *S japonicum* infections.^40^ Excessive Th1 polarization, however, is detrimental to the host, as seen in IL‐4 and IL‐4/IL‐10 double–deficient mice which had a higher mortality than wild‐type mice infected with *S mansoni*.^23^, ^28^ The shift to the chronic Th2 granulomatous response in *S mansoni* infections is considered to be driven by toxic elements of the soluble egg antigens (SEA),^41^ and only recently has an analogous *S japonicum* SEA compound been identified.^42^ Cells recruited to the granuloma primarily include neutrophils, eosinophils, monocytes, lymphocytes and epithelioid cells.^29^ The role of individual Th2 cytokines in granuloma formation and fibrosis have been shown to be different in that IL‐4 or IL‐13 can both generate granuloma formation, while IL‐13 alone is the dominant pro‐fibrotic cytokine in this disease.^43^, ^44^ The Th2‐dominated granulomatous response is in turn regulated by the lesser Th1 response as demonstrated by the fact that in vivo neutralization of IFN‐γ or IL‐12 results in larger granulomas and more extensive fibrosis.^45^, ^46^ In addition, T regulatory cells (Tregs) have been shown to modulate and downregulate granuloma size over the course of chronic infections in mice.^47^, ^48^

Looking more in detail, it is clear that schistosome granuloma composition varies temporally and between species. Early experiments of *S mansoni* hepatic granuloma composition seem to conflict depending on host species.^19^, ^32^ These studies agree, however, that at the granuloma's peak and over the duration of development, the predominant cell type is eosinophils. At 16 days post‐intravenous injection of *S mansoni* eggs in mice, eosinophils account for 50% of the granuloma, with the remainder constituting mostly of macrophages and neutrophils.^32^ In *S japonicum* on the other hand, during the early stages of granuloma formation, the cells in the abscess‐like lesions are predominantly neutrophils in the majority of host species—mice, hamsters, rhesus monkeys and Aotus monkeys.^19^, ^38^, ^49^ As *S japonicum* infection progresses, early experiments showed that late‐stage granulomas in mice are characterized by a decrease in macrophages and neutrophils and an increase in lymphocyte‐like cells and plasma cells on the periphery of periportal inflammation.^32^ The presence of plasma cells suggests local antibody production, and necrotic lesions with polymorphonuclear cells suggest antibody‐antigen complex‐mediated reactions.^50^ Fundamentally, neutrophils were the most predominant cell type in the advanced *S japonicum* granulomas, and they were often degranulated when in contact with the egg shell, with occasional evidence for intraovular immune reactions.^19^

Taken together, these early experiments demonstrate how granuloma formation in *S japonicum* infection contain a significantly higher ration of neutrophils and acute inflammation, compared to those induced by *S mansoni* which are predominantly composed of eosinophils.^19^, ^36^, ^49^

### 5.1 Neutrophils

**FIGURE 1:**
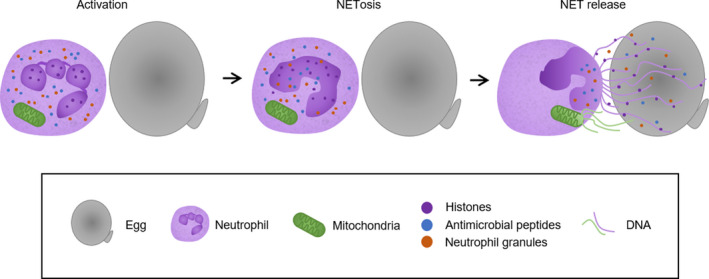
Neutrophil extracellular traps (NETs) in response to *Schistosoma japonicum* eggs. Neutrophils become activated when in contact with *S japonicum* eggs and undergo NETosis—a form of cell death that can leave the neutrophil still viable due to the release of mitochondrial DNA instead of nuclear DNA. NET structures are released, comprised of a DNA backbone with attached histones, antimicrobial peptides and neutrophil granules and enzymes including neutrophil elastase (NE), myeloperoxidase (MPO), lactoferrin and gelatinase among others

As neutrophils are the predominant cell type in the initial inflammatory infiltrate in lesions of primary infections,^51^ and more so in *S japonicum* granulomas compared to *S mansoni*, it is important to understand their function. Neutrophils are the first responders during inflammation and are important for functional innate immunity.^52^ However, they can also trigger host tissue damage when producing proteases and cytotoxins such as reactive oxygen species.^53^ Neutrophils respond to pathogens through a variety of mechanisms, including degranulation, phagocytosis, or via the production of neutrophil extracellular traps (NETs).^54^ NETs have been found to be involved in a variety of responses, involving infection, autoimmunity and cancer,^55^, ^56^, ^57^ and represent a component of neutrophil activity in schistosomiasis infections^58^ (Figure 1).

Neutrophils have been demonstrated to promote damage during schistosomiasis, acting as a major cause of hepatic necrosis and liver damage in the acute stage of *S japonicum* infection as well as progenitors for hepatic fibrosis 2 weeks after implantation of *S japonicum* eggs in mice.^59^ Furthermore, neutrophils were shown to localize to the core of *S japonicum* granulomas through the expression of the neutrophil chemoattractant S100A8, resulting in the production of proinflammatory cytokines and chemokines, including IL‐1α, IL‐1β, IL‐6, TNF, CCL3 and CXCL1, and contributing to overall local tissue damage.^58^ Interestingly, while neutrophils in the granuloma core enhanced inflammation, those on the periphery of the granulomas were found to degrade collagen, the principle component of fibrotic granulomas, introducing a modulatory role for neutrophils in the pathology.^60^

The importance of neutrophils in granuloma formation is evidenced in neutrophil‐depleted *S japonicum*‐infected mice, where hepatic granulomas were augmented over the course of infection^59^ suggestive of a regulatory capacity of neutrophils. Moreover, an *S japonicum* egg protein, SjE16.7, was identified to specifically recruit neutrophils to the site of deposition in vivo,^61^ therefore implicating parasite‐derived factors in modulating neutrophil activity. Such activity includes the employment of NETs after just 4 hours of incubation with *S japonicum* eggs in vitro, suggestive of NET stimulated cell lysis and not vesicular‐mediated responses.^58^ Furthermore, NETs were not produced after interactions with excretory/secretory (ES) proteins from the eggs, suggesting that neutrophil interactions with egg surface or an insoluble factor are required for NET stimulation. Still, NETs were found to not directly kill the egg, but it is suggested that they may restrict further passage.^58^ Taken together, the available data suggest that neutrophils have a crucial role in *S japonicum* granuloma formation and associated pathology.

With regard to *S mansoni* granulomas, neutrophils are not the predominant cell type although they are present at low numbers. Additionally, no NETs are formed by *S mansoni* stimulated neutrophils^60^ despite the presence of egg shell epitopes such as Lewis X carbohydrate epitope^62^ and the ability of the host plasma glycoprotein von Willebrand factor (vWF) to bind to the egg shell surface.^63^ *S mansoni* eggs have been found to secrete a chemokine binding protein (smCKBP), also synonymous with the SEA glycoprotein interleukin‐4‐inducing factor from schistosome eggs (IPSE/α‐1), that interacts with host chemokines and their activity.^64^ Interestingly, smCKBP was found to bind to the neutrophil chemokine CXCL8, inhibiting chemotaxis and neutrophil infiltration in vivo, while not altering the activity of the eosinophil chemoattractant CCL11, which may in part explain the differences in neutrophil/eosinophil granuloma composition in these infections.^64^

### 5.2 Eosinophils

While the eosinophil response to *S mansoni* eggs is much more pronounced than that of neutrophils, the principal role of eosinophils during schistosomiasis is still not fully understood. It remains uncertain whether they act as major effector cells against the worms, or mediators in tissue homeostasis favouring the establishment of the parasites, or merely as facilitators of tissue remodelling and debris clearance during infection.^65^ However, in vitro experiments clearly demonstrated that eosinophil activation can occur through antibody‐dependent cytotoxicity to exert damage on *S mansoni* parasites^66^, ^67^ and that degranulation products such as Major Basic Protein‐1 (MBP‐1) are primary eosinophil mediators in this activity.^65^

Eosinophils constitute around 70% of *S mansoni* egg granulomas in mice 16 days post‐injection, with neutrophils accounting for approximately 10%.^68^ Among the number of chemokines that facilitate eosinophil migration, CCL3‐deficient mice had reduced hepatic granuloma size, fibrosis and eosinophil peroxidase activity, and antibody neutralization of CCL17 and CCL22 both reduced granuloma size and eosinophil recruitment in *S mansoni*‐infected mice.^69^, ^70^, ^71^

Although eosinophils are present in the granulomas of *S mansoni* and *S japonicum* in different proportions, removal of the cell type by administration of anti–IL‐5 antibodies have little effect on granuloma size and fibrosis,^72^ and eosinophil depletion in mice infected with either *S mansoni* or *S japonicum* resulted in slightly smaller granulomas, but had no overall effect on hepatic fibrosis or histology.^72^, ^73^ And although evidence suggests eosinophilic activity was associated with limiting pathology in human *S mansoni* infections,^74^ experiments using *S mansoni* in two different eosinophil ablation mouse models showed no effect on hepatic fibrosis, granuloma size or number.^75^ It should be noted that the absence of the high‐affinity IgE receptor, FcεRI, on mouse eosinophils^76^ may explain the lack of eosinophil‐related effects in murine models.^77^ On the whole, the involvement of eosinophils in *S mansoni* (and *S japonicum* to an extent), granuloma formation and pathology progression is evident but not fully clear, and further investigations are required in order to fully understand their role in pathology.

### 5.3 T cells

T cells are essential for both the generation of granulomas and for the cellular composition of the granuloma, with some differences apparent between the schistosome species.^41^, ^77^ During early experiments, it was observed that granulomas in *S mansoni*‐infected hamsters showed a lymphoid periphery in addition to the initial eosinophil rich infiltration and epithelioid cell centre.^19^ Similar findings were observed in *S japonicum* hepatic granulomas in late‐stage mouse models, with lymphoid cells on the periphery of lesions and periportal inflammation.^32^ In vivo and in vitro studies of *S mansoni*‐infected mice demonstrated the recruitment of SEA‐sensitive splenic CD4^+^ T lymphocytes that were retained in the granuloma and subsequently migrated into the circulation as the infection developed.^78^ Comparisons between athymic mice with *S mansoni* or *S japonicum* infections showed a complete lack of granulomas in *S mansoni* infections and reduced morbidity judged by organ weight, portal pressures and reticuloendothelial activity,^79^ whereas athymic mice with *S japonicum* infections had granulomas present, but they were smaller than controls and had less fibrosis.^80^, ^81^

CD4^+^ Th cells have important roles in many parasitic infections, and as previously discussed, Th1 and Th2 subsets have important roles in schistosomiasis. However, to date, several other types and subsets of T cells have also been identified and classified into several distinct phenotypic classifications, including Th9, Th17 and Tregs.

IL‐9 was initially considered a Th2 cytokine; however, IL‐9 and IL‐4 are rarely produced by the same T cell and Th9 cells are now deemed a unique IL‐9 producing subset of CD4^+^ T cells, characterized by the expression of the transcription factors PU.1 and IRF‐4.^82^, ^83^, ^84^ Not only has it been demonstrated that the level of IL‐9 expression in the liver is significantly increased in chronically infected *S japonicum* mice compared to uninfected controls, but changes in the levels of PU.1, Th9 cells and IL‐9 production all correlate with egg granuloma inflammation.^85^ Li et al^86^ found that although anti‐IL‐9 monoclonal antibody (mAb) neutralization had limited effect on liver inflammation in *S japonicum‐*infected mice, it did reduce total collagen deposition. In addition, levels of IL‐9 increased quicker than IL‐4 in *S japonicum*‐infected mice, indicating a potential role in regulating early stage fibrosis and suggesting that IL‐9 may be a possible target in *S japonicum* hepatic fibrosis modulation.^85^ Similarly, *S mansoni*‐infected C57BL/6 mice showed elevated IL‐9 levels in liver and spleen.^87^ Acute *S mansoni* infections in transgenic mice constitutively expressing IL‐9 were markedly normal with respect to host response; however, in chronic infections, an increased Th2 phenotype was observed, leading to a mortality of 86% at 10 to 12 weeks p.i. in transgenic mice compared to just 7% in wild‐type mice.^88^ Nevertheless, in human *S mansoni* infections, there was no observed correlation of IL‐9 levels with the level of pathology.^89^ These findings suggest that Th9 cells may be involved in the immunopathology of murine schistosomiasis; however, in human infections, the picture is less clear and further studies are required to clarify its role.

Th17 cells are a subset of CD4^+^ T lymphocytes characterized by production of IL‐17. Neutralization of IL‐17 resulted in the inhibition of hepatic inflammation in *S mansoni‐*infected C57BL/6 mice,^90^ while infections in high pathology‐associated CBA mice displayed high levels of IL‐17 and severe hepatic necrosis, which was ameliorated with anti‐IL‐17 mAbs.^90^ Similar results were identified in *S japonicum*‐infected C57BL/6 mice, where neutralizing IL‐17 decreased inflammatory cytokines and reduced recruitment of neutrophils, resulting in improved clinical outcomes.^91^ It, therefore, appears that IL‐17 has a notable contribution in disease severity and clinical resolution in both *S mansoni* and *S japonicum* infections.

Last, the regulation of schistosomiasis infection is carried out by a number of cell types, most notably T regulatory cells which can be categorized into inducible Tregs (iTregs) that increase during infections, and natural Tregs (nTregs) that are an endogenous form of these cells.^92^ The full role Tregs play in *Schistosoma* spp. infections is controversial, partly due to the ambiguities of reliable markers and functional assays.^93^, ^94^ Increased frequencies of human Tregs have been described in both *S mansoni* and *S japonicum* infections.^95^, ^96^, ^97^ Murine studies have demonstrated that after infection with *S mansoni*, the percentage of nTregs (CD4^+^ CD25^+^ Foxp3^+^) increased during the chronic stage of disease^47^ but did not seem to regulate CD4^+^ T cells in vivo,^98^ contrasting with the role of iTregs which were involved in regulation via the production of IL‐10.^99^ The adoptive transfer of CD25^‐^ CD4^+^ T cells to B cell–deficient and T cell–deficient mice (RAG‐deficient) resulted in an increase in liver pathology and mortality during *S mansoni* infection, suggesting an important regulatory role for Tregs during murine infection.^100^ Moreover, Treg involvement in murine colonic granulomas near the site of infection has been shown to be dynamic over the course of the disease.^101^ During chronic stages of infection, there was an approximate fourfold increase in CD4^+^ CD25^+^ Foxp3^+^ Tregs in colonic granulomas, evidenced by an increase in the intestinal homing markers CD103^+^ and CCR5^+^. These colon‐recruited CD4^+^ CD25^+^ Tregs were found to be strong modulators of Th2 responses including granuloma size and eosinophilia, suggesting a role in intestinal schistosomiasis modulation. The enteric environment was also more favourable towards the development of CD4^+^ Foxp3^+^ Tregs over the hepatic environment, which may be why colonic granulomas were modulated faster than those in the liver after the acute phase, and why hepatic granulomas retained their size and cellularity through the chronic phase.^101^

Studies investigating the role of Tregs in *S japonicum* infection found that the CD4^+^ CD25^+^ Tregs recruited in murine models had decreased activity when treated with anti‐CD25 mAbs.^102^ Mice treated with anti‐CD25 mAbs had decreased IL‐10, increased IFN‐γ and a lower worm burden, suggesting that *S japonicum* induces an altered Treg‐modulated immune response that would otherwise elicit a skewed response to target the parasites. Beside CD25, CD4^+^ CD25^+^ Tregs can express markers such as cytotoxic T lymphocyte–associated protein 4 (CTLA‐4),^103^ a key molecule expressed on the surface and in the cytoplasm of peripheral CD4^+^ CD25^+^ Tregs and instrumental in their development and activation.^103^, ^104^ Mice treated with mAbs against CD25 and/or CTLA‐4 showed substantial reductions in worm burdens and egg production^105^ via the impairment of Treg function, allowing for an overactive immune response—demonstrated by increased IFN‐γ, IL‐4, IL‐5 and IL‐10—targeting the worms and eggs, but at the cost of greater pathology shown by larger hepatic egg granulomas.

As discussed previously, *S japonicum* infections are associated with dampened DTH responses, which is in part due to Treg activity. The *S japonicum* HSP‐60–derived immunomodulatory molecule SJMHE1 was shown to inhibit DTH reactions in a mouse model through the induction of CD4^+^ CD25^+^ Tregs.^106^ The lessened inflammation in the DTH reaction by SJMHE1 treatment was associated with a decrease in the inflammatory cytokines IL‐12 and TNF‐α and an increase in the anti‐inflammatory cytokines IL‐10 and TGF‐β1.^35^ Taken together, these findings demonstrate a range of roles that Tregs appear to play in murine *S japonicum* infections.

Human studies have reported that circulating Tregs are increased during *S mansoni* infection, with approximately 40% of patients exhibiting high or very high percentages of circulating Tregs, and treatment with Praziquantel resulting in a reduction of circulating Tregs as well as their expression of CD45RO, a marker associated with T‐cell memory and suppressive activity.^95^ Interestingly, in a study of human *S japonicum* infection, blood and spleen Treg levels were found to be higher in individuals with severe hepatosplenic disease; however, Treg migration to tissues was reduced via impaired CXCR3 expression, suggesting that splenic Tregs unable to migrate to the inflamed liver tissue leads to aggravation of liver disease.^96^ As such, T regulatory cells are clearly involved in controlling pathology in both experimental and human schistosomiasis.

### 5.4 Hepatic Stellate Cells (HSCs)

As discussed above, fibrosis during schistosomiasis infections is largely driven by IL‐13, with IL‐13 knockout mice demonstrating reduced fibrosis.^107^ Hepatic stellate cells (HSCs) are liver resident cells that primarily store vitamin A when quiescent, but when activated by liver damage transdifferentiate into myofibroblasts. These cells are a predominant contributor to collagen production in both *S mansoni* and *S japonicum* infections.^108^, ^109^, ^110^ Characteristics associated with quiescent HSCs are lipid droplet retention and increased expression of peroxisome proliferator‐activated receptor gamma (PPAR‐γ),^111^, ^112^ while activated cells are associated with decreased lipid retention, increased fibrogenesis gene expression, and the increase of stress fibre expression including α smooth muscle actin (α‐SMA) and collagen (Col1A1).^113^

During schistosomiasis, IL‐13 typically activates HSCs to transdifferentiate into myofibroblasts^109^; however both *S mansoni* and *S japonicum* can inhibit this process. *S mansoni* eggs were able to reverse HSC transdifferentiation to return them back to their quiescent state, characterized by HSCs displaying decreased expression of α‐SMA and Col1A1, decreased stress fibre staining, and increased lipid retention when compared to cells cultured without eggs,^113^ supporting the theory that fibrosis is host driven.

Similarly, HSCs cultured with eggs of *S japonicum* reduce their expression of α‐SMA and Col1A1, but in contrast they do not display lipid droplet storage nor have an increased PPAR‐γ expression.^114^ The results were the same for HSCs that had direct contact with the *S japonicum* eggs and those at a distance, suggesting an egg excreted factor causing the effect. Moreover, HSCs in this setting have increased proinflammatory cytokine expression such as CCL2, IL‐6 and MMP9,^114^ proposing a role for HSCs in granuloma development and that both host‐ and parasite‐derived factors contribute to pathology. The variations observed in HSC activation in *S mansoni* and *S japonicum* infections may therefore contribute to the differences seen in overall pathological outcome.

### 5.5 Macrophages

Macrophages (also known as Kupffer cells when liver resident) make up 30% of cells in *S mansoni* liver granulomas. They arise from recruited monocyte‐derived cells and play a role as both mediators and effectors of granuloma formation.^115^, ^116^, ^117^ However, although information on macrophage population involvement in *S japonicum* granulomas is limited,^77^ some known key differences are discussed here.

Antigen‐presenting cells such as macrophages carry out the presentation of egg antigens to CD4^+^ T lymphocytes, leading to the production of various cytokines and chemokines involved in mediating the host response, as well as recruiting additional inflammatory cells.^115^, ^118^ Moreover, macrophages facilitate collagen synthesis through various mechanisms, contributing to the fibrosis‐related pathologies discussed above.^119^, ^120^ Depending on the surrounding cytokine environment macrophages can be activated to become either classically activated macrophages (CAM or M1) or alternatively activated macrophages (AAM or M2), with CAMs stimulated by IFN‐γ, IL‐12 and IL‐18, whereas AAMs are dependent on stimulation from cytokines such as IL‐4 and IL‐13 inducing expression of arginase‐1 (Arg‐1) and Fizz‐I.^121^

During experimental schistosomiasis infections, too extreme polarization to either Th1 or Th2 result in severe pathology, morbidity and death.^112^ In strongly Th1‐polarized IL‐4/IL‐13–deficient mice, Arg‐1 fails to be expressed^122^, ^123^ and such Th1‐polarized mice display increased inducible nitric oxide synthase (iNOS) activity and increased mortality. Furthermore, *S mansoni*‐infected Arg‐1–deficient mice displayed increased hepatic fibrosis and mortality. As such it appears that Arg‐1 expressing AAMs have a role in controlling pro‐inflammatory pathology, while promoting the generation of fibrotic pathology.^123^, ^124^, ^125^ Macrophage activation to CAMs is seen in the acute stage of *S japonicum* infections, transitioning to the development of AAMs during the chronic stage,^126^ the latter of which is regulated in part by IL‐33 stimulating IL‐5 and IL‐13 secretion.^127^ It was observed that egg ES products polarized macrophages to M2b—a subset of AAMs—in a Toll‐like receptor (TLR)‐2–dependent manner, and TLR‐2^‐/‐^ mice displayed altered cytokine profiles and smaller granulomas with less fibrosis compared to wild type mice.^128^ These findings illustrate some of the similarities of responses that can be found to infections of both species, despite more research carried out with *S mansoni* parasites.

In addition to the role of AAMs in fibrogenesis, they have also been demonstrated to be involved in fibrotic resolution and are a main source of anti‐fibrotic MMP13.^77^, ^129^, ^130^ Macrophages in a cholestatic rat liver model were shown to recruit neutrophils as additional collagenase‐producing cells, during the resolution of extracellular matrices.^131^ Gene expression markers of AAM, such as Chi3l3 and Retnlg, were upregulated in the core and periphery of *S japonicum*‐induced hepatic granulomas, akin to neutrophil infiltration in granulomatous lesions,^60^ suggesting a role for macrophages at granuloma peripheries in inducing matrix degradation and stimulating a neutrophil inflammatory response, resulting in both hepatic fibrotic resolution and pathology. Furthermore, the *S japonicum* egg‐specific protein SjE16.7 was described to recruit neutrophils to the site of egg deposition, however, it was also found to be a potent macrophage activator through both chemotaxis and cytokine production and pathology was greatly reduced after in vivo blockade.^132^ As such there appears to be multiple roles for macrophages in granuloma formation and fibrogenesis related to specific cell infiltrates that may affect the variations seen in *Schistosoma* spp. pathology.

## 6 CONCLUDING REMARKS

Schistosomiasis is a debilitating and global disease that imposes approximately 3 309 000 disability‐adjusted life years (DALYs), a fundamental measure of disease burden.^133^ *S mansoni* and *S japonicum* are the main aetiologies for the intestinal forms of the disease and are crucial to target if the disease is to be attenuated. As there is currently just one treatment available (Praziquantel), which does not protect against reinfection, the likelihood of parasite drug resistance is high and mechanisms of pathology must be fully understood in order to generate new treatments to reduce disease burden.^134^

**FIGURE 2:**
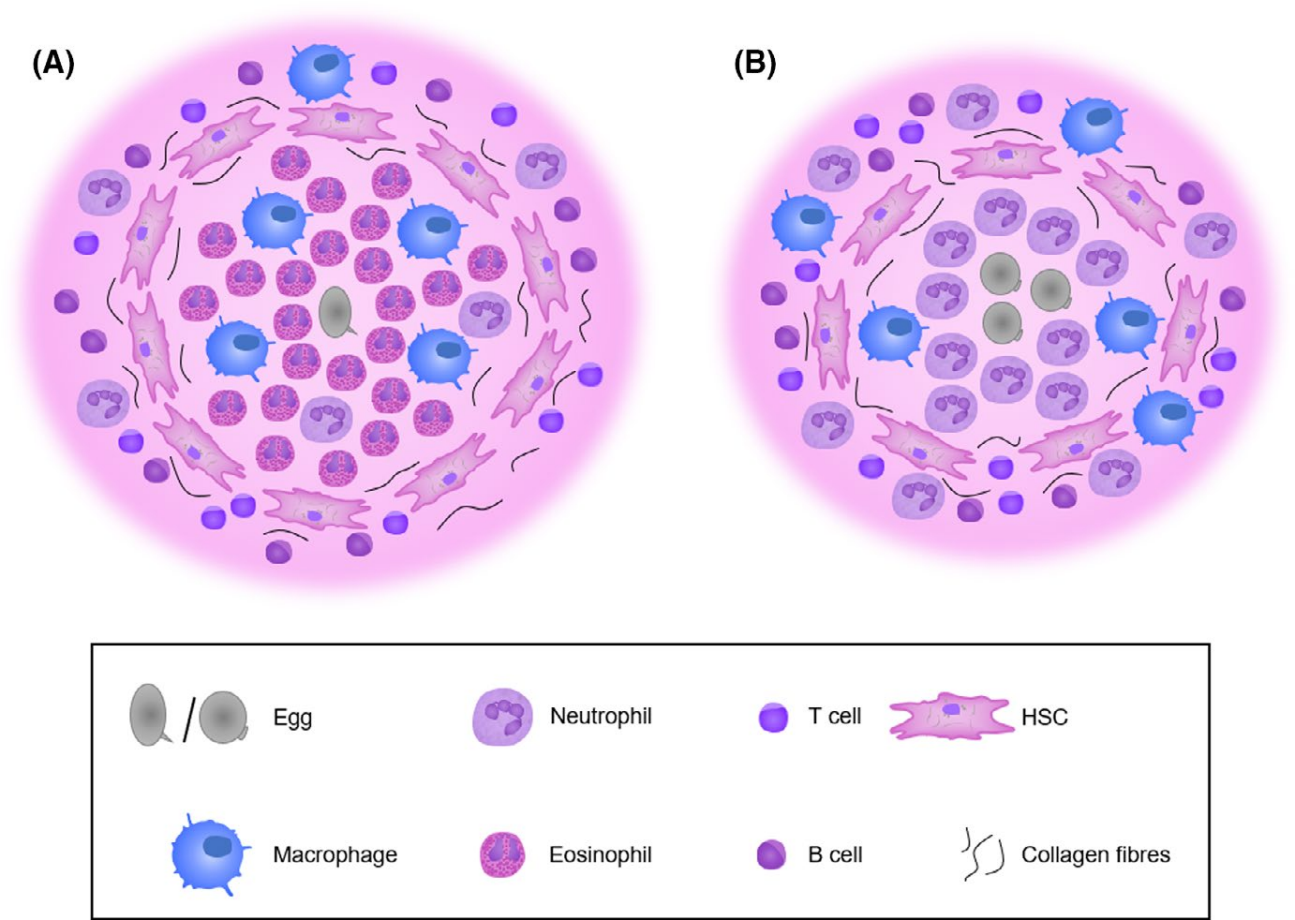
Illustration of granuloma composition in *S mansoni* (A) and *S japonicum* (B) and the predominant cell types. *S mansoni* granulomas are typically larger, induced by a single egg and have an eosinophil dominant core. *S japonicum* granulomas may be formed around a cluster of eggs and are dominated primarily of neutrophils. HSC, hepatic stellate cell

The principal cause of chronic pathology is the egg‐induced host granulomatous response, and as discussed here, there are both clear differences and similarities in the mechanisms and regulations of the host response between *S mansoni* and *S japonicum* (Figure 2). Despite some features pointing towards *S mansoni* being the more pathological disease—such as eliciting generally larger single‐egg granulomas and displaying the ability to decrease gut stromal integrity and remodel the vasculature of Peyer's patches^135^ there is now much scientific evidence to suggest that the overall pathology induced by *S japonicum* is the more severe of the two species at all stages of infection and in multiple host species.^19^, ^36^, ^37^, ^38^ As we have discussed here, even subtle differences in the initiation of various cytokines and chemokines affect downstream effectors with regard to cellular recruitment, thus contributing to the different levels of pathology seen between *S japonicum* and *S mansoni* infections. Some of the mechanisms behind the increased pathogenesis of *S japonicum* are discussed here, from the role of neutrophils in host‐mediated pathology, to the parasite‐derived compounds modulating the host response. Together, this difference between the two primary causes of intestinal schistosomiasis highlights the need to examine all elements, as well as consolidate where there are similarities, in order to fully understand this disease.

## CONFLICT OF INTEREST

The authors explicitly state that they have no conflict of interest to disclose.
